# Nosocomial Transmission of *Plasmodium falciparum* Malaria, Spain, 2024

**DOI:** 10.3201/eid3109.250920

**Published:** 2025-09

**Authors:** Jesús L. Gómez Perales, Antonio García Mendoza, M. Teresa Gutiérrez Amares

**Affiliations:** Puerta del Mar Hospital, Cádiz, Spain (J.L. Gómez Perales, M.T. Gutierrez Amares); Torrecárdenas, Almería, Spain (A. García Mendoza).

**Keywords:** malaria, Plasmodium falciparum, vector-borne infections, acquired malaria, parenteral transmission, parasites, aseptic technique, cross-contamination, nuclear medicine, lead shield, procedural guidelines, patient safety, radiopharmaceutical administration, Spain

**To the Editor:** We wish to express our concerns regarding the recent Research Letter by Liroa Romero et al. (*1*). The authors’ identification of the lead shield as the transmission source is inferred from the exclusion of other routes, procedural sequence, parasitemia in the previous patient, and genotypic similarity. The absence of demonstrated contamination of the equipment means this finding remains indirect. An unaddressed alternative route could include contamination through blood on gloves if gloves are not changed between patients (*2*).

Nevertheless, assuming their hypothesis is correct, the authors propose a transmission mechanism supported by a video (https://youtu.be/2OW9g2tiBjc). However, this video appears to depict a deviation from current good radiopharmacy practice guidelines (*3*). Specifically, inserting an unsealed syringe—without a sterile needle or Luer-lock cap—into the lead shield poses a serious contamination risk (*4*). Furthermore, the video omits critical earlier and later procedural steps that are essential for fully identifying potential cross-contamination points. To illustrate the complete process in line with good radiopharmacy and best injection practices (*5*), we have prepared an explanatory video (https://youtu.be/5wGFH6GGe8M). The risk for blood contamination arises after administration, when the needle is discarded and the unsealed syringe is withdrawn from the shield. Therefore, the contents of the syringe with the next dose would only be exposed at the same late stage, after the contents have already been injected. Cross-contamination before injection would be plausible only if an unsealed syringe were inserted into an already contaminated shield before the injection itself, as depicted in the authors’ video.

We contend that strict aseptic technique, rather than equipment disinfection alone, is paramount to preventing such incidents. Adopting the authors’ reasoning would imply that each nuclear medicine department would need to stock several lead shields equivalent to their maximum daily dose capacity, which presents serious logistical challenges in routine clinical settings.
